# Perceived discrimination among people of colour and minorities in European neurosurgery: A survey-based study

**DOI:** 10.1016/j.bas.2025.105627

**Published:** 2025-10-10

**Authors:** Silvia Hernández-Durán, Andrew F. Alalade, Claudio Cavallo, Doortje Engel, Makinah Haq, Tijana Ilic, Katrin Rabiei, Fozia Saeed, Gargi Sarmath, Nikolaos Syrmos, Yu-Mi Ryang, for the European Association of Neurosurgical Societies' Diversity in Neurosurgery Committee

**Affiliations:** a Department of Neurosurgery, Rostock University Medical Center, Schillingallee 35, 18057, Rostock, Germany; b Department of Neurosurgery, Royal Preston Hospital, Lancashire Teaching NHS Foundation Trust, PR2 9HT, United Kingdom; c University of Central Lancashire, Preston, PR1 2HE, United Kingdom; d Department of Neurosurgery, Universitätsspital Zürich, Rämistrasse 100, Zürich, 8091, Switzerland; e Health Department of Canton Grisons, Switzerland; f GKT School of Medical Education, King's College London, United Kingdom; g Clinic for Spinal Surgery, Spinal Center of the Catholic Hospital Bruederhaus Koblenz, Universitätsstraße 1, Koblenz, 56070, Germany; h Institute of Neuroscience and Physiology, Sahlgrenska Academy Gothenburg, Bla straket 1, Gothenburg, 413 46, Sweden; i Department of Neurosurgery, Leeds Teaching Hospitals Trust, Beckett Street, Leeds, LS9 7TF, United Kingdom; j Department of Neurosurgery, Imperial College Healthcare NHS Trust, S Wharf Rd, London, W2 1NY, United Kingdom; k Aristotle Universtiy of Thessaloniki, Egnatia, Thessaloníki, 541 24, Greece; l Dept. of Neurosurgery & Center for Spine Therapy, HELIOS Klinikum Berlin-Buch, Schwanebecker Chaussee 50, Berlin, 13125, Germany

**Keywords:** European neurosurgery, Discrimination, Diversity, Minorities

## Abstract

**Background:**

European neurosurgery is becoming increasingly diverse. However, professionals from minority and migrant backgrounds may encounter discrimination. Empirical data on these experiences remain limited.

**Objective:**

This study assesses the prevalence and nature of perceived discrimination among minority and migrant neurosurgical professionals across Europe. It evaluates whether migration status, self-identified minority status, and gender are associated with differences in perceived discrimination.

**Methods:**

A 30-item online survey was disseminated through the European Association of Neurosurgical Societies. Items addressed demographic details, professional background, perceived discrimination, reporting behavior, and institutional policies. Perceived discrimination was measured using a five-point Likert scale assessing frequency of discrimination based on ethnic origin or immigrant background. Mann-Whitney U tests were employed for group comparisons due to the ordinal nature of the data. Significance was set at p < 0.05.

**Results:**

Among 105 respondents, 42 (40 %) were classified as migrants, and 18 (17 %) self-identified as ethnic or religious minorities. Unexpectedly, migrant status, minority status and female gender did not statistically correlate with self-reported experience of discrimination. Institutional structures to address discrimination were often unknown or absent.

**Conclusion:**

Migration status and self-identified minority status or gender, was most strongly associated with perceived discrimination. These findings highlight the importance of understanding invisible forms of bias and the complex intersection of identity, nationality, and institutional culture.

## Highlights

• First survey on perceived discrimination in European neurosurgery.
• 40 % of respondents were migrants; 17 % identified as minorities.
• No link between discrimination scores and gender or minority status.
• Institutional anti-discrimination structures often unknown or absent.

## 1 Introduction

The professional landscape of European neurosurgery is increasingly shaped by demographic shifts and international mobility (Saeed et al., 2023). Despite this growing diversity, structural barriers to inclusion persist, particularly for individuals from minority or migrant backgrounds.

Empirical studies have highlighted the structural challenges faced by underrepresented minorities in neurosurgery. In the United States, racial and ethnic minorities account for a disproportionately small fraction of neurosurgery trainees and faculty. Black physicians make up only 4.8 % and Hispanic physicians 5.8 % of neurosurgery residents, compared to their respective 13.4 % and 18.5 % shares of the general population (Asfaw et al., 2022). Faculty representation is even more limited, suggesting that retention and promotion are additional barriers in the neurosurgical pipeline. Importantly, the disparities are not solely numeric but compounded by the lived experiences of exclusion, lack of mentorship, and diminished access to informal professional networks (Borden et al., 2022; Saeed et al., 2023).

Intersectionality—particularly at the nexus of race and gender—further intensifies the barriers encountered by certain groups. Black women, for example, remain almost entirely absent from U.S. academic neurosurgery. As Bryant et al. (Bryant et al., 2021; Odell et al., 2019) detail, the history of black women in neurosurgery is not simply one of delayed entry, but one marked by ongoing systemic exclusion. Even among institutions that espouse meritocratic ideals, black female neurosurgeons report experiencing a “layering” of inequity that extends from residency recruitment through to faculty promotion.

Although most research focuses on the U.S., parallel issues are surfacing in Europe. Borden et al. (2022) demonstrate that conventional metrics—such as gender alone—fail to capture the complexity of diversity in neurosurgery. They advocate for a multidimensional framework that accounts for intersecting factors like ethnicity, nationality, socioeconomic status, and educational background. This approach is especially critical in European contexts, where legal and cultural norms often render ethnic identity invisible (Simon, 2007; Turton and González Ferras, 1999).

Saeed et al. (2023) provides the first systematic mapping of ethnic and racial representation in neurosurgical leadership across Europe. Drawing on data from 39 countries, the study found that underrepresented minorities (URM) presence among departmental heads remains low in most European nations, although not uniformly so. Countries with high immigration rates, such as the United Kingdom and Germany, showed relatively higher levels of minority representation in neurosurgical leadership. By contrast, some regions—including several Balkan states—had no URM representation at all. This study emphasizes the correlation with broader national demographic patterns and migration histories. Importantly, European neurosurgery does not appear to be uniformly exclusionary, but rather to exhibit structural disparities that require context-specific responses.

Taken together, these studies underscore the urgent need for comprehensive, context-specific research into how discrimination, underrepresentation, and institutional inaction manifest in neurosurgical environments. The present study seeks to address this gap by analyzing self-reported experiences of discrimination among neurosurgeons across Europe. This is the first study on self-reported discrimination in European neurosurgery. Particular attention is given to how these experiences intersect with migration status, gender, and perceived institutional support.

## 2 Methods

### 2.1 Study design

This was a cross-sectional, observational survey study conducted in accordance with the Checklist for Reporting of Survey Studies (CROSS) guidelines. The design aimed to collect and analyze self-reported perceptions and experiences of discrimination among neurosurgical professionals in Europe, focusing on URM groups.

### 2.2 Questionnaire development

The survey instrument was created by the Diversity in Neurosurgery Committee of the European Association of Neurosurgical Societies (EANS). The final questionnaire consisted of 30 items, including single-choice, multiple-choice, and Likert-scale questions. These were grouped into sections covering: (1) demographics and professional status, (2) migration and minority identity, (3) perceived experiences of discrimination (race, ethnicity, religion), (4) institutional mechanisms and reporting behavior, and (5) open-ended qualitative feedback.

The questionnaire was developed collaboratively by the committee members. It was reviewed internally for clarity and face validity. While formal validation was not performed, all items were designed to align with existing literature on workplace discrimination in healthcare. Similarly, no formal pre-testing or pilot was conducted involving an external participant sample; however, informal feedback from committee members and affiliated neurosurgical trainees was used to refine wording and question flow. The full questionnaire is available as supplementary electronic material (SEM).

### 2.3 Target population and sampling

The target population comprised neurosurgeons and neurosurgical trainees practicing or training in Europe. Eligibility criteria included: current or past professional engagement with a European neurosurgical institution, fluency in English (the survey language), and voluntary consent. There were no exclusion criteria based on nationality, ethnicity, or level of seniority.

A convenience sampling strategy was employed. The survey was distributed electronically via the EANS mailing list, national neurosurgical societies, institutional contact points, and professional social media networks. This means several thousand neurosurgeons and trainees from 39 countries were invited to participate.

Given the anonymized and open dissemination strategy, no formal sample size calculation was performed.

### 2.4 Survey administration

The survey was hosted on Google Forms and was accessible online for a continuous period of three months from June through August 2023. Respondents could complete the survey from any location. Only one submission was permitted per IP address, and participation was voluntary and anonymous. No identifying information was collected.

### 2.5 Ethical considerations

The study was conducted in compliance with the Declaration of Helsinki. Formal ethical approval was not required under EU GDPR guidelines due to the anonymized nature of the survey and the absence of clinical or patient-identifying data. All participants were informed at the beginning of the survey about the purpose of the study, the voluntary nature of participation, and their right to withdraw. Submission of the survey implied informed consent.

### 2.6 Statistical analysis

All statistical analyses were performed using Python (SciPy, Pandas, Seaborn). Perceived discrimination scores, measured on a 5-point Likert scale, were treated as ordinal variables. Group differences—based on gender, minority identification, and migration status—were assessed using the Mann-Whitney *U* test. Categorical variables were summarized as frequencies and percentages. Figures were created with visme.

## 3 Results

A total of 105 individuals completed the survey. Of these, 66 participants identified as male (62.9 %) and 36 as female (34.3 %), while three respondents preferred not to disclose their gender. Age ranged from 24 to 65 years. Seniority of the respondents within the field of neurosurgery was as follows: 9 department chairs, 4 professors, 11 associate professors, 44 consultants, 35 residents/fellows. Respondent countries are summarized in Table 1.

**Table 1:** Country of practice of the respondents in alphabetical order.

Country of practice	Number of respondents
Albania	1
Austria	2
Belgium	1
Bulgaria	1
Croatia	2
Czech Republic	2
Estonia	1
Finland	5
France	2
Germany	28
Greece	6
Israel	2
Italy	5
Lithuania	1
Netherlands	5
Norway	1
Poland	3
Portugal	1
Romania	3
Serbia	1
Spain	3
Sweden	1
Switzerland	4
Turkey	4
United Kingdom	12
Ukraine	2
Other	3

### 3.1 Migration and minority background

**Fig. 1:**
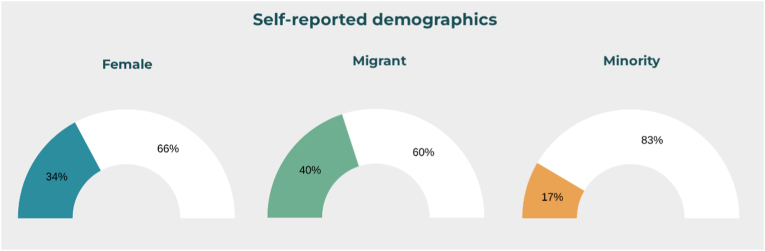
Proportion of respondents identifying as female, migrant or minorities in their countries of practice.

Of the total respondents, 42 individuals (40.0 %) reported being born in a different country from the one in which they currently practice or received neurosurgical training. These participants were categorized as migrants. Eighteen individuals (17.1 %) self-identified as belonging to a minority group in their current country of residence based on ethnic, religious, or cultural criteria (Fig. 1). These two classifications were not mutually exclusive, and some participants identified with both.

Those who reported having migrated were presented with a checklist of possible motivations for leaving their country of birth. The most commonly selected reasons were the pursuit of better-paying jobs, improved career potential, and access to higher-quality training, each cited by 61.9 % of migrant respondents. Family-related reasons such as reunification were indicated by 23.8 %, and 19.0 % cited personal preference. Reasons related to escaping persecution were reported by 11.9 % of migrants, including persecution on religious, ethnic, political, or gender-related grounds. A smaller portion reported structural or educational barriers in their home countries, with 2.4 % indicating they could not obtain residency or citing better access to affordable education.

When asked why they chose their current country of training or practice, half of all migrants (50.0 %) cited better academic opportunities. Around one-third of respondents selected better hands-on opportunities (35.7 %) and improved economic conditions or remuneration (35.7 %). Better work-life balance was selected by 28.6 %, while 23.8 % cited family decisions. Cultural familiarity, such as proximity to one's heritage or language proficiency, was cited by 16.7 %. Additional reasons—each selected by 2.4 % of respondents—included asylum status, institutional openings, and tuition-free university access.

### 3.2 Self-reported experiences of discrimination

Among respondents, 11.7 % reported experiencing discrimination on the basis of skin colour “often,” and 10 % reported experiencing it “very often.” For discrimination based on ethnic origin or immigrant background, 16.7 % respondents reported experiencing it “often,” and 23.3 % selected “very often.” Discrimination related to religion or religious beliefs was reported “often” by 1.7 % respondent and “very often” by 11.7 % respondents.

Of the respondents who indicated experiencing discrimination, the majority selected multiple forms through which it had manifested. Of those who experienced discrimination, 75.0 % reported unequal treatment in comparison to peers of non-minority background. Derogatory comments from superiors were reported by 35.0 %, matched by an equal number who indicated being overlooked for promotions or career advancement. Derogatory comments from peers were reported by 30.0 %. Assignment of “unattractive” cases was identified by 26.7 %.

### 3.3 Denouncing of discrimination

The majority of participants did not report discriminatory behaviors experienced by themselves (73.3 %). These reports did not yield any consequences for the perpetrators in 45.8 %, and instead led to negative consequences for the reporting party in 12.5 % of cases. Reasons for not reporting discriminatory behaviors were fear of negative impact on ones own career in 27.8 % of cases, perceived lack of institutional support in 26.6 %, and feeling of futility in 22.8 %.

While 37.9 % participants have designated offices at their institutions to address any kind of discrimination, most did not report having any initiatives to address discrimination in the workplace (49.5 %).

### 3.4 Strategies moving forward

Increasing awareness through open discussion was selected by 67.0 % of participants as a strategy to address discrimination in neurosurgery. Other suggestions including highlighting the importance of diversity in major neurosurgical associations (58.3 %) and mentoring programs (56.3 %).

### 3.5 Group differences

Migrant status, minority status and female gender did not statistically correlate with self-reported experience of discrimination.

## 4 Discussion

While gender equity has become a visible and often-quantified dimension of diversity efforts in neurosurgery (Demetriades et al., 2020; Hernández-Durán et al., 2021; Lambrianou et al., 2022; Murphy et al., 2021; Wang et al., 2022; Weiss et al., 2023; Wolfert et al., 2019), racial and ethnic inclusion remains critically underexplored. In the United States, where race is frequently a central axis in discussions of structural inequality, the literature nevertheless reveals substantial neglect. A systematic review by Wang et al. (2022) analyzing U.S.-based neurosurgical diversity initiatives found that only two of fifteen included studies reported data specific to URM representation, and none systematically addressed the experiences, perceptions, or institutional barriers faced by these groups. Despite evidence that URM individuals comprise less than 15 % across medical education, residency, and faculty roles in U.S. neurosurgery (Asfaw et al., 2022; Bryant et al., 2021), targeted efforts to address racial inclusion remain rare and underreported. Much of the existing work focuses on gender diversity, leaving race—and particularly the intersection of race with gender or socioeconomic background—largely unexamined.

This omission is mirrored, though not identically replicated, in the European context. Saeed et al. (2023) conducted a cross-sectional analysis of ethnic minority representation in neurosurgical leadership across 39 European countries, marking the first attempt to systematically assess this issue at a continental level. Their findings revealed wide national variation – from countries with significant URM representation in leadership roles to countries with no URM representation – reflecting the complexity of European national identities and demographic make-ups, alongside with the historical population shifts that have characterized the continent in the past century (Ball et al., 2022a; Simon, 2007; Turton and González Ferras, 1999).

The current study, complementarily, is the first one to evaluate perceived discrimination of URM in European neurosurgery. Quite unexpectedly, it did not reveal any statistically significant differences in the perception of discrimination based on ethnic/racial grounds for migrants, minorities and/or women. While this might initially appear counterintuitive given the scope of anecdotally reported exclusionary experiences, it aligns with broader findings from European public opinion research suggesting that exclusionary attitudes may not map neatly onto discrete demographic categories. For example, Coenders et al. (2021) demonstrated that while nationalist sentiment varies considerably across European countries, these attitudes are remarkably stable within countries and are not reliably predicted by short-term shifts in immigration or socioeconomic conditions. Instead, nationalism correlates more strongly with long-standing individual factors—such as lower education, higher religiosity, and perceived ethnic threat—than with actual demographic change.

This broader attitudinal stability may offer insight into our findings. Discriminatory behavior in professional environments like neurosurgery may stem less from acute identity-based targeting and more from ambient cultural climates shaped by deep-rooted, specific nationally inherent beliefs about belonging and hierarchy. In such settings, the experience of exclusion may be widely distributed and variably perceived, rather than confined to specific groups. Our inability to detect statistically significant differences does not preclude the presence of a structurally exclusionary environment—it may instead reflect a uniform exposure to subtle, normalized forms of bias.

The absence of statistically significant differences in reported discrimination across key identity groups does not necessarily mean that it is absent. This finding of our study may be further contextualized by recent research on racism and intersectionality in Europe. Ball et al. (2022b) argue that European societies often operate within a framework of "postracialism," in which race is culturally and politically silenced, and whiteness is maintained as an implicit normative standard. In this context, racism is frequently reframed as cultural incompatibility, which may obscure how discriminatory practices are actually experienced. Their review of European labor market studies suggests that intersectional forms of bias—especially those involving ethnicity, religion, and gender—manifest in complex and context-dependent ways. For example, while Arab men were found to be disproportionately targeted in high-profile jobs in Scandinavian countries, Muslim women experienced heightened exclusion when occupying visible or high-status roles in academic institutions. Such patterns suggest that discrimination in Europe does not always follow linear or additive logics based on demographic categories alone. This aligns with our finding that perceived discrimination was not significantly stratified by gender or minority status and supports the interpretation that bias in professional environments like neurosurgery may be more structurally diffused, context-specific, and normalized than captured by traditional subgroup comparisons.

### 4.1 Limitations

Our study may have failed to identify statistically significant differences because of the group sizes and the relatively low response rate. This is undoubtedly a bias and the main limitation of this work. The sample size and the convenience sampling aggravate these limitations. Numerous studies have shown that individuals frequently refrain from reporting discriminatory experiences due to fear of retaliation, reputational harm, or damage to professional relationships—concerns that are especially salient in high-status, hierarchical fields such as neurosurgery (Cortina and Magley, 2003; Sue et al., 2007). Subtle forms of discrimination such as microaggressions may also be dismissed as too ambiguous to justify a formal complaint, while pervasive exposure to bias can lead to normalization or emotional fatigue, reducing the likelihood of action over time (Cortina and Magley, 2003; Nielsen et al., 2009; Sue et al., 2007). These factors collectively underscore the complexity of this topic and suggest that the underreporting of discrimination in our sample likely reflects structural and cultural deterrents rather than a genuine absence of discriminatory experience. Another limitation and an indirect result are that the topic was not of great interest of the overall target population, which leads to a decreased response rate (Groves et al., 2004). Some individuals probably have responded because they find the topic important for society, although it does not form an issue to their daily business. Others, that responded that they did not report discrimination in the workplace might have seen this questionnaire as an opportunity to raise their issue by responding in hope of indirect call for societal changes.

## 5 Conclusion

This study demonstrates that perceived discrimination in European neurosurgery is low. Contrary to expectations, self-identification as a minority was not linked to increased discrimination scores, and gender alone did not predict discrimination perceptions. These findings highlight the importance of understanding invisible forms of bias and the complex intersection of identity, nationality, and institutional culture. Improved communication, better visibility of support structures, and continued empirical monitoring are essential for building a more inclusive neurosurgical workforce in Europe.

## Declaration of competing interest

The authors declare that they have no known competing financial interests or personal relationships that could have appeared to influence the work reported in this paper.
