# Supplementary material for: Perceived discrimination among people of colour and minorities in European neurosurgery: A survey-based study
Source: Brain Spine. 2025 Oct 10;5:105627. doi: 10.1016/j.bas.2025.105627 (PMC12554926; doi:10.1016/j.bas.2025.105627)
Supplement: Multimedia component 1 [file mmc1.zip › bas_105627_People of Colour and Minorities in European Neurosurgery_V2_mmc1.PDF]

People of Colour and Minorities in European Neurosurgery

Dear participant,
thank you for your interest in the European Association of Neurosurgical Societies (EANS) Diversity in Neurosurgery Committee survey on "People of colour and minorities in European neurosurgery". Through this survey, we aim to identify factors pertaining to discrimination based on ethnic/religious background in European neurosurgery, behaviours related to them, and ways in which they can be addressed.
If you wish to know more about our committee, please visit www.eans.org/page/Diversity_Commitee
Thank you for your participation!
The EANS Diversity in Neurosurgery Committee

* Indicates required question

1. This consent form asks you to allow the researcher to record and view the interview and to use your comments to enhance understanding of the topic. The form also asks your permission to use related observations as data in this study. Participation in this survey is completely voluntary. If you decide not to participate there will not be any negative consequences. Please be aware that if you decide to participate, you may stop participating at any time and you may decide not to answer any specific question. The researcher will maintain the confidentiality of the research records or data. By submitting this form you are indicating that you have read the description of the study, are over the age of 18, and that you agree to the terms as described. *

   Mark only one oval.

   - Yes
   - No

Demographics

2. Age *

   ____

3. Sex *

Mark only one oval.

- ◯ Female
- ◯ Male
- ◯ Prefer not to say
- ◯ Other: ____________________

4. Country of practice *

____

5. Country of training *

____

6. Current position * ◯ Dropdown

Mark only one oval.

- ◯ Resident/Fellow/Trainee
- ◯ Attending/Consultant
- ◯ Associate Professor
- ◯ Professor
- ◯ Department chair

7. Is your country of birth different from your country of training and/or practice? *

Mark only one oval.

- ◯ Yes *Skip to question 8*
- ◯ No *Skip to question 13*

Immigration background

In this section, we would like to know more about why you left your country of birth for your country of practice

8. At what age did you leave your country of birth? *

____

9. Do you hold permanent residency in your current country of practice? *

Mark only one oval.

- ( ) Yes
- ( ) No

10. Do you hold citizenship in your current country of practice? *

Mark only one oval.

- ( ) Yes
- ( ) No

11. Why did you leave your country of birth? (check all that apply) *

Check all that apply.

- [ ] Opportunity. Hope to find better-paying job, better career potential, better training.
- [ ] To escape persecution. Persecution can be due to religious, ethnic, political, gender-related issues, for example.
- [ ] Personal preference.
- [ ] Family reunion/family. Includes following parents/spouse to new country.
- [ ] Prefer not to say
- [ ] Other: ________________________

12. Why did you choose your current country of training and/or practice? (check all that apply) *

Check all that apply.

- [ ] Better academic opportunities.
- [ ] Better hands-on opportunities.
- [ ] Better work-life balance.
- [ ] Family's choice.
- [ ] Cultural proximity to my own.
- [ ] Was granted asylum in this country.
- [ ] For economic reasons, e.g. better job remuneration.
- [ ] Prefer not to say.
- [ ] Other: ______________________

Skip to question 14

13. Do you consider yourself to be a minority in your country, be it for ethnic, religious or other reasons? *

Mark only one oval.

- ◯ Yes *Skip to question 14*
- ◯ No *Skip to question 24*

Discrimination due to minority background

14. Have you experienced discrimination on the basis of skin colour? *

Mark only one oval.

	1	2	3	4	5	
Very	◯	◯	◯	◯	◯	Very often

15. Have you experienced discrimination on the basis of ethnic origin or immigrant background? *

Mark only one oval.

	1	2	3	4	5	
Very	◯	◯	◯	◯	◯	Very often

16. Have you experienced discrimination on the basis of religion/religious beliefs? *

Mark only one oval.

	1	2	3	4	5	
Very	◯	◯	◯	◯	◯	Very often

17. How was this discrimination manifested? (check all that apply) *

Check all that apply.

- [ ] Decreased caseload in comparison to peers of non-minority background.
- [ ] Lower surgical complexity in cases assigned, in comparison to peers of non-minority background.
- [ ] Assignment of "unattractive" cases, in comparison to peers of non-minority background.
- [ ] Assignment of "unattractive" rotations, in comparison to peers of non-minority background.
- [ ] Differences in salary/reimbursement/remuneration.
- [ ] Derogatory comments from peers.
- [ ] Derogatory comments from superiors.
- [ ] Derogatory comments from patients/family members.
- [ ] Overlooked for promotions/career advancement.
- [ ] On-call schedule.
- [ ] Paperwork/administrative work/ward work.
- [ ] Outright verbal abuse.
- [ ] Sexual harrassment.
- [ ] I have not experienced any discrimination.
- [ ] Other: ____________________

18. Did you report any of these behaviours? *

Mark only one oval.

- ( ) Yes *Skip to question 19*
- ( ) No *Skip to question 23*

If you reported discriminatory behaviour

19. To whom did you report these behaviours? *

Mark only one oval.

- ( ) Immediate superior
- ( ) Mentor
- ( ) Head of department
- ( ) Hospital administration - Human resources
- ( ) Hospital/academic leadership
- ( ) Medical/doctors' union/association
- ( ) Local authorities
- ( ) Government authorities
- ( ) Other: ______________________

20. How many times did you file a report? *

____

21. What was the outcome of the reported incidents? (check all that apply) *

Check all that apply.

- [ ] A discussion involving all parties was had.
- [ ] Perpetrators were addressed by superiors/head of department.
- [ ] Perpetrators were officially warned by the hospital authorities.
- [ ] Legal repercussions for the perpetrator.
- [ ] Measures were taken to avoid discrimination (workshops, policies, etc.)
- [ ] No consequences for the perpetrator.
- [ ] Negative consequences for me and my career as a reporter of discrimination.
- [ ] Change of working place due to negative work environment.
- [ ] Other: ______________________

22. If you are a member of a medical union, did you get any support from them when reporting discrimination? *

Mark only one oval.

- ( ) Yes
- ( ) No
- ( ) N/A

Skip to question 26

If you did not report discriminatory behaviour

23. Why did you not report these behaviours? (check all that apply) *

Check all that apply.

- [ ] Fear of retaliation.
- [ ] Feeling of futility.
- [ ] Perceived lack of institutional support.
- [ ] Precedent - previous experiences from others indicating futility/negative consequences/retaliation.
- [ ] Fear of negative impact on career progression.
- [ ] I did not experienceand/or observe any discriminatory behaviour.
- [ ] Other: ______________________

Skip to question 26

Observation of discrimination

24. Did you observe any of these discriminatory behaviours towards a person of colour/member of a minority group at your institution? (check all that apply) *

Check all that apply.

- [ ] Decreased caseload in comparison to peers of non-minority background.
- [ ] Lower surgical complexity in cases assigned, in comparison to peers of non-minority background.
- [ ] Assignment of "unattractive" cases, in comparison to peers of non-minority background.
- [ ] Assignment of "unattractive" rotations, in comparison to peers of non-minority background.
- [ ] Differences in salary/reimbursement/remuneration.
- [ ] Derogatory comments from peers.
- [ ] Derogatory comments from superiors.
- [ ] Derogatory comments from patients/family members.
- [ ] Overlooked for promotions/career advancement.
- [ ] On-call schedule.
- [ ] Paperwork/administrative work/ward work.
- [ ] Outright verbal abuse.
- [ ] Sexual harrassment.
- [ ] I have never observed any discriminatory behaviour.
- [ ] Other: ______________________

25. Did you report any of these behaviours? *

Mark only one oval.

- ( ) Yes *Skip to question 19*
- ( ) No *Skip to question 23*

Skip to question 26

Institutional accountability

26. Does your institution have an office to address any kind of discrimination, independent from human resources? *

Mark only one oval.

- Yes
- No
- I don't know

27. Does your institution have workshops to address discrimination in the workplace? *

Mark only one oval.

- Yes
- No
- I don't know

28. Does your institution conduct any initiatives to address discrimination in the workplace? *

Mark only one oval.

- Yes
- No
- I don't know

29. Does your local medical union conduct any initiatives to address discrimination in the workplace? *

Mark only one oval.

- Yes
- No
- I don't know

Future directions

30. How do you think discrimination towards people of colour/minorities in neurosurgery should be addressed? (check all that apply) *

Check all that apply.

- [ ] Increase awareness through open discussion.
- [ ] Workshops to learn to identify microaggressions in the workplace.
- [ ] Supporting people of colour/minorities in their career advancement through mentorship programs/leadership workshops.
- [ ] Highlight the importance of diversity in major neurosurgical associations, e.g. EANS, WFNS, etc.
- [ ] Webinars on diversity in the neurosurgical workplace.
- [ ] Other: ______________________

Voluntary section

31. I would like to participate in a structured interview about my experiences as a person of colour/minority/immigrant and thus agree to have my data deanonymised. (Please enter E-mail address)

____

This content is neither created nor endorsed by Google.

Google Forms
